# The Emerging Therapeutic Targets for Scar Management: Genetic and Epigenetic Landscapes

**DOI:** 10.1159/000524990

**Published:** 2022-06-13

**Authors:** Sara Amjadian, Sharif Moradi, Parvaneh Mohammadi

**Affiliations:** ^a^Experimental Medicine and Therapy Research, University of Regensburg, Regensburg, Germany; ^b^Department of Stem Cells and Developmental Biology, Cell Science Research Center, Royan Institute for Stem Cell Biology and Technology, ACECR, Tehran, Iran; ^c^Department of Developmental Biology, University of Science and Culture, Tehran, Iran

**Keywords:** Wound healing, Hypertrophic scar, Scar-promoting genes, MicroRNA, Noncoding RNAs

## Abstract

**Background:**

Wound healing is a complex process including hemostasis, inflammation, proliferation, and remodeling during which an orchestrated array of biological and molecular events occurs to promote skin regeneration. Abnormalities in each step of the wound healing process lead to reparative rather than regenerative responses, thereby driving the formation of cutaneous scar. Patients suffering from scars represent serious health problems such as contractures, functional and esthetic concerns as well as painful, thick, and itchy complications, which generally decrease the quality of life and impose high medical costs. Therefore, therapies reducing cutaneous scarring are necessary to improve patients' rehabilitation.

**Summary:**

Current approaches to remove scars, including surgical and nonsurgical methods, are not efficient enough, which is in principle due to our limited knowledge about underlying mechanisms of pathological as well as the physiological wound healing process. Thus, therapeutic interventions focused on basic science including genetic and epigenetic knowledge are recently taken into consideration as promising approaches for scar management since they have the potential to provide targeted therapies and improve the conventional treatments as well as present opportunities for combination therapy. In this review, we highlight the recent advances in skin regenerative medicine through genetic and epigenetic approaches to achieve novel insights for the development of safe, efficient, and reproducible therapies and discuss promising approaches for scar management.

**Key Message:**

Genetic and epigenetic regulatory switches are promising targets for scar management, provided the associated challenges are to be addressed.

## Introduction

**Fig. 1:**
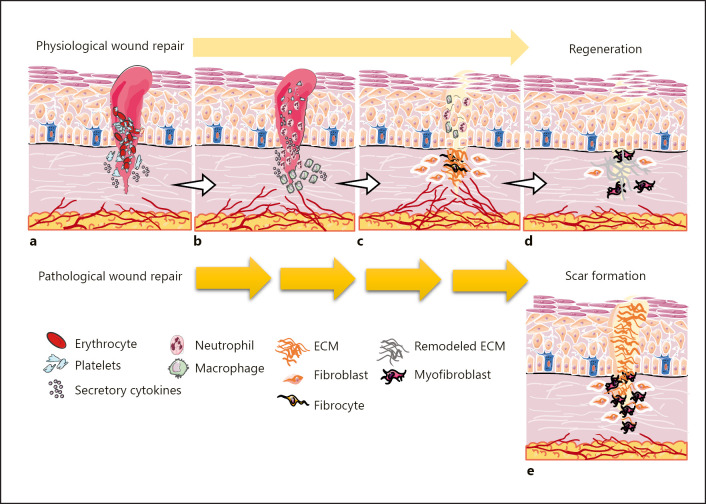
The cutaneous wound healing process, a regenerative or reparative response. The skin tissue response following injuries in gestation and injured fetal tissues is regeneration via a physiological wound healing process including four stages. **a** In the hemostasis phase, platelets are involved in the formation of the blood clot and the release of cytokines required in the inflammatory cell recruitment. **b** In the inflammation stage, neutrophils and macrophages are activated to phagocytosis of pathogens and damaged cells. Moreover, neutrophils secrete cytokines increasing the severity of inflammatory responses and macrophages facilitate the transition from inflammation to proliferation. **c** In the proliferation phase, reepithelialization leads to the covered wound surface, the vascular network is restored, and the provisional matrix is replaced with the granular tissue. **d** In the remodeling, stage fibroblasts differentiate into myofibroblasts, and collagen type III fibers are replaced with collagen type I fibers. **e** Any abnormalities which are leading to delayed repair or enhanced cell responses propel the wounds to pathological or reparative healing accompanied by cutaneous scar formation which is happened in human adults.

As a guard of the body, the skin is constantly exposed to the potential causes of injuries. The normal tissue response following injuries is skin regeneration via the process of wound healing which includes hemostasis, inflammation, proliferation, and remodeling stages [1] (shown in Fig. 1). In the hemostasis phase, a fibrin clot is formed operating as a primary scaffold for the migratory cells. Platelets are the main players in this stage, being involved in the release of required cytokines to recruit the inflammatory cells (Fig. 1a). In the inflammation stage, the recruited neutrophils and macrophages are activated by cytokines to phagocytose pathogens and damaged cells in the wound bed. Neutrophils also secrete cytokines and interleukins (ILs), increasing the severity of inflammatory responses. Thereafter, macrophages facilitate the transition from inflammation to the proliferation phase (Fig. 1b). During the proliferation phase, the wound surface is covered via the migratory, proliferative, and differentiated keratinocytes, which is termed reepithelialization. In this phase, a vascular network is also restored and the provisional matrix is replaced with a granular tissue containing a large number of fibroblasts, granulocytes, macrophages, blood vessels, and collagen proteins (Fig. 1c). Finally, during the remodeling phase, fibroblasts differentiate into α-smooth muscle actin (αSMA)-expressing myofibroblasts, which generate contractile forces to promote wound closure. Moreover, type III collagen fibers, the most abundant component of the extracellular matrix (ECM), are replaced with type I collagen fibers to regenerate a wound with the maximized tensile strength of the skin [1, 2, 3, 4] (Fig. 1d). Noticeably, the regulated transition of different phases in the wound healing process results in optimal skin repair which is a unique ability in fetal injured tissues at early gestation, while any abnormalities lead to the accumulation of a nonfunctional fibrotic tissue which is known as a scar and happened mostly in human adults [5, 6, 7] (Fig. 1e). Cutaneous excessive scars, hypertrophic or keloid, are the major challenges for the patients and physicians since they cause serious health problems such as contractures, functional and esthetic concerns as well as painful, thick, and itchy complications. These issues generally reduce the quality of life physically, mentally, and socially and impose high medical costs [8, 9].

Nowadays, several strategies are used for the prevention and treatment of raised scars, including pressure garments, silicone gels and sheets, corticosteroids, lasers, surgical procedures, etc. Pressure garments restrict the blood flow to the scar area through compression of the local blood vessels, thus limiting blood supply and causing hypoxia of the scar tissue that consequently increase collagenase activity and decrease cohesion between collagen fibers. Pressure therapies also modulate secretion of fibrotic cytokines and growth factors. Mechanical stimulations affect cell apoptosis, migration, proliferation, differentiation, and collagen fiber turnover, resulting in thinning and softening of scar tissues. However, the effective application of pressure garments highly depends on the applied pressure levels. Measuring the exact pressure level is difficult and controversial between theoretical therapeutic and practically observed pressure magnitude. Moreover, early cessation of pressure therapy leads to the contraction and thickening of scar tissues. A recent meta-analysis study examining six randomized trials regarding the use of pressure garments in patients with large burn scars demonstrated no significant difference between pressure garments and no-treatment group [10, 11]. On the other hand, silicone gels and sheets work through increasing the temperature and hydration of the occluded scar. In a Cochrane review, which analyzed 15 controlled trials related to silicone gels and sheets, no evidence was found to suggest that they are superior to alternate or to no-treatment group in preventing or treating scar tissues [12]. Additionally, although corticosteroids suppress inflammation, increase vasoconstriction in the scar, reduce collagen and glycosaminoglycan production, and lessen fibroblast proliferation, side effects such as telangiectasia, hypopigmentation, subcutaneous atrophy, and pain associated with steroid injection restrict the application of this clinical method. Finally, constraints on obtainable skin quantities for skin grafts, the possibility of transplanted tissue rejection, and prevention of infection and excessive tension across the wound raise further complications related to this strategy [12, 13, 14]. Accordingly, conventional approaches are not specific and there is no evidence to support their absolute efficacy. Even if the best of therapies are used, traumatic scarring is unavoidable and the available treatments only help to minimize scarring. Therefore, there is a great requisiteness to establish new methods based on the optimization of wound healing procedure to diminish or prevent dermal fibrosis. This purpose necessitates advances in the understanding of mechanisms, key regulators, and risk factors involved in wound healing [3, 9, 15]. Therapeutic interventions focused on basic science including genetic and epigenetic knowledge are recently taken into consideration as promising approaches for scar management since they have the potential to provide targeted therapies and improve the conventional treatments as well as presenting opportunities for combination therapy.

Thus, in this review, we aimed to highlight the diversity of the major histocompatibility complex (MHC) genes and single-nucleotide polymorphisms (SNPs) determining the susceptibility to scar formation to establish the prognosis and prevention strategies and subsequently personalized medicine. We also depict the genetic network and associated dysregulation in inflammation, granulation tissue formation, apoptosis, and ECM remodeling to providing insights for genetic manipulation to optimize wound healing and block skin fibrosis. Finally, we consider epigenetic alterations including DNA methylations, histone modifications, and noncoding RNAs such as microRNAs and long noncoding RNAs (lncRNAs) as promising therapeutic tools for scar management.

## From Genetic Architecture to Scar Management

The factors driving skin tissue regeneration are strongly associated with regulatory gene networks. Deregulated genes are thus expected to either predispose individuals to cutaneous scarring or directly promote abnormal wound healing and scar formation. Hence, deciphering the association between the genes and aberrant wound healing help introduce efficient therapeutic interventions. This requires identifying genetic variations and causative genes involved in wound healing and scarring as discussed below.

### Genetic Variations and Susceptibility in Scar Formation

Genetic variations are defined as the differences in DNA sequence among people that play an important role in susceptibility to a particular disease. Diversity of the MHC genes and single base-pair substitutions are such genetic causes putting the patients at risk of skin scarring [16, 17]. MHC genes in humans, which are known as human leukocyte antigens (HLAs), impact cutaneous fibrosis via antigen-presenting capacity and immune system regulation. HLA molecules work as the co-inducers of inflammation and cytokine expression affecting the lesions through induction of fibroblast proliferation and collagen deposition. Some particular alleles of the HLA system have a higher propensity to bind antigens, leading to more intense immune responses in predisposed individuals [17, 18]. Studies have shown the increased levels of HLA-DR, HLA-B14, and HLA-Bw16 alleles in individuals with hypertrophic and keloid scar compared to normal tissue [18, 19]. Among HLA-DR alleles, the HLA-DRB*16 allele was reported to predispose individuals to postburn hypertrophic scar formation, while the HLA-DRB1*15 allele increased the risk of keloid tissue formation in Caucasians [17, 20, 21]. Moreover, a positive association of HLA-DQA1*0104, HLA-DQB1*0501, and HLA-DQB1*0503 alleles with keloid diseases was observed in Chinese Han populations. In these patients, HLA class I members such as HLA-A*03, HLA-A*25, HLA-B*07, and HLA-Cw*0802 were also found to be more frequent compared to matched control group [17, 22, 23].

Single base-pair substitutions known as SNPs are the other variations in the human genome where a single nucleotide is replaced by another one in at least 1% of the population [24, 25]. Several studies have shown that SNPs alter the function of genes involved in wound healing and scarring, as they either increase or reduce the susceptibility of individuals to cutaneous fibrosis. Sood et al. [26, 27, 28] identified SNPs R163Q, rs56234898, and rs11136645 in melanocortin 1 receptor (*MC1R*), protein tyrosine phosphatase nonreceptor type 5 (*PTPN5*), and CUB and sushi multiple domains 1 (*CSMD1*) genes, respectively. SNP R163Q in *MC1R* is associated with an increased probability of postburn hypertrophic scarring. Given that the melanocortin signaling pathway affects the skin scarring through decreased dermal fibroblast proliferation, anti-inflammatory, and anti-fibrotic effects, SNP R163Q-induced MC1R loss of function might enhance the risk of inflammation and fibrosis [28, 29]. On the contrary, SNP rs56234898 in *PTPN5* reduces cutaneous scarring. PTPN5 known as striatum-enriched protein tyrosine phosphatase is a tyrosine phosphatase with a binding motif for mitogen-activated protein kinases particularly p38. SNP rs56234898 alters the gene function and potentially enhances the inhibitory effect of striatum-enriched protein tyrosine phosphatase on P38 in myofibroblasts or other inflammatory cells, resulting in decreased severity of scarring [26, 30]. SNP rs11136645 in *CSMD1* is also associated with a decreased hypertrophic scar in white subjects. CSMD1 is an inflammatory regulator involved in the development of the nervous system and wound healing process [26, 31], albeit little is known about its role in wound repair. *CSMD1* and SNP rs11136645 seem to reduce fibrosis through regulation of complement activation, neuroinflammation, and/or TGF-β1 signaling pathway [27, 32]. In another study, Teng et al. [33] reported SNPs rs181924090, rs151091483, rs183178644, and rs141156594 in the sirtuin 3 (*SIRT3*), myosin heavy chain 8 (*MYH8*), HUS1 checkpoint clamp component B (*HUS1B*), and rotatin (*RTTN*) genes, respectively, in the Chinese Han Population. In this study, rs181924090, rs151091483, and rs183178644 were identified as new potential SNPs associated with keloid formation, especially related to tumor behaviors of keloids, whereas rs141156594 was known as a new SNP involved in the ECM formation in wound healing [33]. Furthermore, Ogawa et al. [34] found SNP rs8032158 in the neural precursor cell expressed developmentally downregulated protein 4 (*NEDD4*) gene that enhances the possibility of keloid formation. rs8032158 contributes to excessive cell proliferation and matrix deposition and could be a biomarker for the prevention of keloid formation.

In summary, MHC alleles and SNPs are intrinsic factors causing differences among individuals in terms of susceptibility to disease, the capacity to promote disorders, and the resistance to drugs and therapies. Therefore, identification of such genetic indicators may provide the possibility to predict the risk and progression of traumatic scarring as well as characterization of scar types following skin injuries. Genetic variations are not the only causative factors for scar formation. However, their potential importance in the development of appropriate and personalized strategies for the prevention or treatment of cutaneous scars cannot be ignored. To achieve this goal, genetic screening to discover such variations and understanding their roles might be helpful, which would need certain tools and technologies, and further properly designed studies in the future.

### Causative Genes in Scar Formation

Following skin injuries, many genes are up- or downregulated to modify the wound environment. However, deregulated expression of the genes involved in this critical process can promote abnormal wound healing and scarring. Identification of these genes offers the opportunity to manage the different stages of wound healing and modulate fibrosis through gene therapy to convert scarring injuries into scar-free repaired wounds [35]. Various genes can be considered for scar-free wound healing such as those contributing to inflammation, proliferation, apoptosis, granulation tissue formation, and ECM remodeling (Table 1) as discussed below.

#### The Genes Responsible for Inflammation

An optimized wound healing relies on a proper level of inflammatory factors to ensure that cellular responses are presented in a coordinated manner. Severe injuries in the skin barrier dramatically increase cytokine and chemokine production, which may dictate tissue fibrosis. Using gene expression profiling of cytokines from keratinocytes of burned patients, Gragnani et al. [36] showed that IL-8 is highly expressed in large skin burns. IL-8 works as a chemoattractant for neutrophils, basophils, T lymphocytes, and fibroblasts and serves to restore the epidermal hemostasis. IL-8 is also associated with stimulated angiogenesis of endothelial cells, amplified keratinocytes mitosis, and regulated production of metalloproteinases-9 (MMP-9) in keratinocytes [37, 38]. Therefore, IL-8 is a prerequisite to promote wound healing normally. However, the related excessive secretions may intensify cellular responses, thereby driving cutaneous scarring.

Similarly, overexpression of IL-1α, IL-1β, IL-6, and tumor necrosis factor-α (TNF-α) has been observed in fibrotic tissues [39]. IL-1α regulates the activation of keratinocytes, fibroblasts, and endothelial cells that attract circulating leukocytes and induces the expression of chemokines which in turn persist the inflammation [40, 41]. On the other hand, IL-1β upregulates decorin in dermal fibroblasts, working coordinately with each other to activate and enhance peripheral blood mononuclear cells (PBMCs) homing potential toward the wound bed. In the next induction pick, transforming growth factor-beta (TGF-β) dictates a fibrocystic transition from localized PBMC [42]. Expectedly, deregulated levels of IL-1α and IL-1β result in the development of skin scarring. Thomay et al. [43] demonstrated that downregulation of IL-1 signaling improves the quality of wound healing and lessens fibrosis. Cutaneous wounds in IL-1 receptor knockout mice represent reduced levels of collagen and improved architecture of restored skin compared to wild-type animals [43]. Moreover, IL-6 is a modulator of inflammation promoting the pro-inflammatory function of T cells and macrophages and also is required for the timely resolution of wound healing. Studies show that downregulation of IL-6 in hypertrophic and keloid scars results in an improved scar score [44]. TNF-α is also the other pro-inflammatory cytokines promoting the inflammation and infiltration of immune cells into the wound bed. TNF-α also stimulates BMP2 and its receptor in keratinocytes, thereby provoking the epithelial-to-mesenchymal transition (EMT) and cell migration [45, 46, 47, 48]. Hence, the enhanced expression of TNF-α leads to prolonged inflammation and continuous EMT, driving the hypertrophic scar or keloid formation.

On the contrary, IL-10 is known as an anti-inflammatory factor and regulates the expression of fibrogenic cytokines such as TGF-β contributing to ECM formation. IL-10 has a protective role against excessive collagen deposition and αSMA expression and modulates the survival of precursor endothelial cells in damaged tissue [49, 50, 51, 52]. Peranteau et al. [53] indicated that injection of lentiviral vectors expressing IL-10 at the wound site in an adult murine model of scar formation decreased the quantities of pro-inflammatory mediators and consequently inflammation. Injected lenti-IL-10 vectors in the wound bed also showed normal collagen deposition and restoration of dermal architecture [53]. Importantly, recombinant human IL-10 in phase II randomized controlled clinical trial reduced scar formation and improved scar appearance [49] demonstrating a translational potential of this cytokine in clinical application for scar therapy in humans, as yet requires further considerations.

#### The Genes Responsible for Proliferation, Apoptosis, and Granulation Tissue Formation

An accelerated secretion of cytokines and chemokines in response to the injured skin leads to stimulating the growth factor production which is directly affecting the cellular events of the wound healing process particularly cell proliferation, differentiation, ECM deposition, and apoptosis. Platelet-derived growth factor (PDGF) and insulin-like growth factor-1 (IGF-1) are critical players produced to augment wound repair [54, 55, 56, 57, 58]. Barker et al. [59] demonstrated that ex vivo genome editing of mouse fibroblasts for sustained secretion of PDGF increases the speed and quality of wound healing, at least in part, through enhanced vascularization. Additionally, Amiri and colleagues [60] demonstrated that IGF-1 gene transferring into fibroblasts and transplanting them to full-thickness wounds of rat models resulted in an increased number of keratinocytes, enhanced organization of granular tissue, and improved wound contracture. Gene delivery of IGF-1 via adenoviral vectors to chronic wounds of mice also accelerates epithelial gap closure, promotes granulation tissue formation, and enhances neovascularization, reported by Balaji et al. [61]. However, the proper level of expressed PDGF and IGF-1 should be maintained since they correlate to the signaling pathways such as TGF-β, ERK, and kinase cascades which consequently drive the sequential events leading to skin fibrosis [62, 63].

Similarly, TGF-β is a pivotal growth factor required to support successful wound repair. TGF-β signaling is activated through integrin crosstalk and regulated by a negative feedback loop mechanism. Hence, any abnormalities in TGF-β signaling pathways cause skin tissue disorders [64, 65]. Studies have revealed that high and consistent expression of TGF-β-1, TGF-β-2, or TGF-β receptors in the wounded skin results in collagen accumulation, impairing tissue architecture and function. Thus, TGF-β signaling could be a therapeutic target for scar management. Washio et al. [66] represented that gene silencing of *TGF-β1* at promoter region via synthetic pyrrole-imidazole polyamide in a rat model inhibits the induction of growth factors and ECM-encoding mRNAs, and limits the number of spindle-shaped fibroblasts. Wang and colleagues [67] showed that downregulation of the TGFβRI gene via siRNA in a rabbit model of hypertrophic scar reduces the ECM deposition of scar tissue and the expression of CTGF and αSMA mRNAs. Application of bioengineered matrix delivering RNAi against TGFβ1 by Liu and colleagues [68], on the skin defects of a pig model, revealed the inhibition of TGFβ1, collagen, and αSMA expression, and also the similar structure of regenerated skin compared to the healthy skin tissue. Finally, concurrent silencing of TGF-β and COX-2 via siRNA in mouse models implanted with human skin graft activated cell apoptosis and reduced skin fibrosis, which was demonstrated by Zhou et al. [69], and in phase II human clinical trial [70]. On the contrary, TGF-β3 plays anti-fibrotic roles promoting collagen degradation via upregulation of matrix metalloproteinases (MMPs) and reducing collagen type I deposition via limited fibroblastic differentiation [37, 71]. Therefore, the ratio of TGF-β3 to TGF-β1 or TGF-β2 expression is a determining factor to propel physiological or pathological wound healing [71]. It is worth noting that TGF-β translation is induced by an immediately upstream regulator, P311. P311 also supports collagen deposition, normal scar formation, and tensile strength of newly formed tissue preventing scar dehiscence [72, 73]. Cheng et al. [72] depicted that *P311* gene deficiency in knockout mice significantly reduces collagen deposition and tensile strength, whereas delivery of the *P311* gene through lentiviral vectors corrects the defects. On the other hand, overexpression of P311 during deep wound repair may contribute to hypertrophic scar formation, and lentiviral transfer of shRNA against P311 downregulates collagen and hydroxyproline content in scars of wild-type mice [72]. Therefore, regarding the importance of TGF-β signaling pathway during wound healing, and promising results obtained in related preclinical and clinical studies, it appears that TGF-β signaling possesses the potential to be considered as a therapeutic target in pharmaceutical marketing, as the associated challenges are addressed.

In an optimized wound repair, the formation and restoration of the blood vessel network supporting oxygen and nutrients for the involved cell populations is a crucial step regulated by vascular endothelial growth factor (VEGF). VEGF binds to the VEGF receptor and stimulates the activation of downstream kinases to improve endothelial cell survival, proliferation, and migration [74]. Additionally, homeobox genes and related proteins such as Hox-A5 and Hox-A9 are capable of affecting angiogenesis. Hox-A5 reduces the expression of pro-angiogenic genes such as VEGFR-2, ephrin A1, hypoxia-inducible factor 1 subunit alpha (HIF-1), and COX-2 in the endothelial cells, and increases the expression of antiangiogenic genes such as thrombospondin-2, whereas Hox-A9 enhances the transcription of VEGF [75]. Studies indicate that gene delivery of VEGF by adeno-associated viruses accelerates wound healing through well-structured granulation tissue formation and greater vascularization as well as increased epithelium regeneration and neo-angiogenesis in the rat models [76]. However, the high level of VEGF results in hypertrophic and keloid scarring; thus, a proper expression of VEGF could be a therapeutic target for controlling dermal fibrosis [74, 77].

The programmed cell death is also considered as a critical mechanism converting the cell-rich granular tissue to fibrous tissue in a normal process of wound healing. Thus, the tissues with deregulated apoptotic genes such as *p53*, *Bax*, *Bcl-2*, and upstream factors including slug and secreted frizzled-related protein 2 (SFRP2) direct an aberrant repair leading to the raised scar formation [78, 79]. The factors involved in programmed cell death can be remarkable candidates for gene therapy and subsequently wound repair with less or without scar formation. Shi and colleagues [80] revealed that gene delivery of *p53* through adenoviral vectors and inhibition of *Bcl2* gene by siRNA in a rabbit ear scarring model results in smaller and flatter scars compared to control wounds that were injected with adenoviruses carrying shRNA against *P53*. Additionally, the more neatly arranged and thinner structure of collagen fibers during the scar remodeling process is resulted from the upregulation of *P53* and downregulation of *Bcl2* genes, while using shRNA against *P53* leads to the more disordered structure and denser collagen fibers [80]. Hence, targeting apoptosis pathways may be a promising approach to propel the wound to heal normally and preventing scarring, as the balance is maintained with the survival and proliferation signaling. Expectedly, this requires further designed preclinical and clinical studies and consideration of all challenges in the future.

#### The Genes Responsible for ECM Remodeling

An important event in the remodeling phase of cutaneous wound healing is the proteolytic degradation of ECM to restore functional tissue architecture. MMPs and tissue inhibitor metalloproteinases (TIMPs) are responsible for the degradation and renewal of ECM in the late phase of wound repair. The wound healing procedure is potentially affected by MMP-1, −2, −3, −8, −9, and −13. MMP-1, −8, and −13 work as collagenases I, II, and III, respectively, degrading the fibrillar collagens. The created segments are then denatured to produce gelatins and degenerated by the gelatinases such as MMP-2 and −9 [81, 82, 83]. The activity of MMP is controlled by TIMPs. TIMPs bind to and inhibit the MMPs functions, thereby preventing the ECM degradation and enhancing the total ECM proteins [84]. TIMP family contains several protease inhibitors such as TIMP-1, −2, and −3, of which TIMP-1 and −2 work as effective inhibitors of MMP-1 and −2, respectively, and TIMP-3 inhibits the activity of MMP-1, −2, −3, −9, and −13 [83, 85]. Expectedly, deregulated expression of MMPs and their inhibitors leads to imbalanced collagen matrix deposition and degradation, which consequently drives excessive cutaneous scarring. Simon et al. [86] revealed that the continuous expression of TIMP-1 in the activated keratinocytes may contribute in the enhanced dermal thickness and created hypertrophic scars. Arakawa, Neely, and their colleagues reported the reduced expression and activity of MMP-1 and −9 promoting the hypertrophic and keloid scar formation [87, 88]. Imaizumi et al. [89] reported a high level of MMPs such as MMP-2 in the hypertrophic and keloid tissues, with this explanation that the overexpressed MMPs may not only be inadequate to overcome the excessive signals that promote scarring but also help in keloid extension to adjacent normal skin through ECM degradation.

Furthermore, procollagen-lysine2-oxoglutarate 5-dioxygenase 2 (PLOD2) and decorin are the other regulators playing important roles in the rearrangement phase of wound healing. PLOD2 mediates the formation of collagen cross-linkages such as pyridinoline. The pyridinoline cross-links are associated with the collagen molecules' resistance against degradation by MMPs. Therefore, the increased level of PLOD2 and pyridinoline cross-link content give rise to less collagen degradation and eventually the accumulation of fibers, as observed in hypertrophic and keloid scars [83, 90, 91]. On the other hand, decorin regulates the tensile strength of the skin through interaction with collagen molecules and noncollagenous proteins such as fibronectin [83]. This protein binds to and neutralizes TGF-β, thus minimizing the inductive effects of TGF-β on collagen, fibronectin, and GAG production [6]. In dermal ECM, decorin is produced normally, while downregulated in the wound healing process following severe injuries [64, 83]. Danielson and colleagues [92] demonstrated that reduced expression of decorin in mice gives rise to thin and fragile skin, abnormal morphology, and nonuniformity in the axial mass distribution of collagen fibrils, and reduced collagen-bound proteoglycans which potentially reduce the tensile strength of the skin and increase the incidence of scarring.

Overall, cutaneous wound repair is a complex and multifactorial process regulated by various cells, cytokines, growth, differentiation, and apoptotic factors. Any abnormalities in the expression and function of such regulators result in impaired wound healing and possibly scarring. It seems that gene therapy is a novel therapeutic approach for optimization of wound healing and scar management since gene delivery can be accomplished for a limited time until healing and also a limited area of interventions. On the other hand, transient effects due to the short half-life and difficulties to properly penetrate the wound bed and reach the targeted cells prevent the topical application of cytokines and growth factors [93, 94]. For successful genetic manipulations, there are yet several issues that must not be ignored. First, the healing of the cutaneous wound is mediated by different cell types including macrophages, mast cells, keratinocytes, fibroblasts, myofibroblasts, endothelial cells, and more, which have been targeted for different drugs and therapeutic strategies [95].

Hence, the appropriate selection of targeted cells with aberrant behavior provoking the initiation and progression of scarring is a crucial step in this regard. Keratinocytes and fibroblasts are the most common cells that have been considered in different animal models through ex vivo and in vivo gene transferring [76]. However, keratinocyte stem cells due to the ability to generate the sufficient storage of functional epithelial cells and fibroblasts from the reticular layer of the dermis due to their further potential for scar formation [96] may be more exciting candidates for genetic modifications. Second, numerous genes which are associated with inflammation, proliferation, apoptosis, granulation tissue formation, and ECM remodeling as well as their processing enzyme coding genes or their receptor-related genes can be targeted for manipulations. However, spatiotemporal intervention is another key step since some regulators may have a dual role in the wound healing process. For instance, TNF-α is known as both inflammatory and apoptotic factors, overexpressed in the early and downregulated in the late phases of wound healing, and promotes scarring [97, 98]. Similarly, the increased expression of MMPs in the inflammation stage provokes keratinocyte migration and prolonged inflammation, while its downregulation in the late stages leads to the accumulation of ECM [81, 99]. Thus, temporal and spatial targeting of such factors to reverse their expression could affect the outcomes of gene therapy. Third, many aforementioned mediators are essential for optimized wound healing. Thus, a proper level of manipulations should be exerted to ensure that the cellular responses occur in an orchestrated manner and do not impede normal repair. Regulatory inducible systems including the tetracycline, ecdysone, and rapamycin inducible systems, which their activities are stimulated by pharmacological molecules, and also gene-activated matrix such as engineered biomaterial scaffolds which support the sustained and long expression of desired gene are the strategies considered to regulate the expression of transgene [100]. Fourth, the selection of an appropriate strategy is crucial to fulfilling the safe, targeted, and efficient delivery. Genetic interventions can be performed through viral or nonviral approaches and depend on the purpose of therapies. Although they are contributed to immunogenicity, viral gene deliveries include 70% gene therapy clinical trials [101]. Retroviral gene delivery of *LAMB3* into the keratinocytes by De Luca et al. [102] for epidermolysis bullosa patients is one of the most prominent gene therapies offering promises for treatment of other skin disorders as well. Moreover, regarding wound repair, injection of adenoviruses encoding PDGFB in patients with venous leg ulcers, demonstrated in phase-1 clinical study, represents the reduced size of wounds [103]. On the other hand, lipid-based nanoparticles, polymeric nanoparticles, antisense oligonucleotide, siRNAs, and physical methods are such nonviral delivery systems attracted great attention in preclinical studies and clinical trial for treatment of skin disorders [101].

Phase II clinical trials for dual-targeted siRNA STP705 and RXI-109 also indicate the potential of these small RNAs as next-generation medicine. STP705 affects the expression of TGF-β1 and COX-2 mRNAs simultaneously, and RXI-109 efficiently targets CTGF mRNA, attenuating the cutaneous fibrosis [69, 104, 105, 106]. With the emergence of site-specific nuclease editing tools such as ZFNs, TALENs, and clustered regularly interspaced short palindromic repeats (CRISPR)-Cas9 system, it seems that gene therapy would be a promising therapeutic approach in the future to genetically modify resident wound bed cells, both transiently and permanently, albeit being still in infancy [106].

## From Epigenetic Knowledge to Scar Management

Gene transcripts are regulated based on the open or compact patterns of specific gene loci under a physiological or pathological condition. Such regulations can be conducted by the mechanisms encompassed in epigenetic knowledge. Epigenetic regulation has been recently investigated as the potential mechanism for changing the cell behavior and phenotype during wound healing and scar maintenance, thus promising novel targets for scar treatments [107]. DNA methylation, histone modifications, and noncoding ribonucleic acid alterations are epigenetic regulations affecting the cell fate in wound repair as discussed below.

### The Dynamic State of DNA Methylation and Histone Modifications during Scar Formation

DNA methylation is an important chromatin modification responsible for the long-term changes in the cell programming by which the gene transcription is generally repressed. The maintenance of DNA methylation patterns entails the DNA methyltransferase (DNMT) activity upon cell replication. Thus, aberrant expression of DNMTs is always correlated with abnormal DNA methylation and consequently human diseases such as fibro-proliferative disorders [107, 108]. For instance, DNMT1 is indicated to be increased significantly in both hypertrophic and keloid scars compared with normal skin. This protein plays an important role in the regulation of collagen synthesis and ECM deposition by scar fibroblasts and is strongly associated with the keloid expansion beyond the initial wound [108, 109, 110]. The evidence shows that the inhibition of DNMT1 activity via the inhibitors such as 5-aza-2-deoxycytidine represses the proliferation of hypertrophic and keloid fibroblasts, increases the ratio of cells in the G0/G1 phase of the cell cycle, and enhances apoptosis. Additionally, upon the intervention of 5-aza-2-deoxycytidine, the expression of pro-fibrotic cytokines including TGF-β1 is decreased, while the expression of SMAD7, as an inhibitory downstream factor of the TGF-β1 signaling pathway, is increased [108].

The genetic potential of DNA can be affected by histone modifications as well. Acetylation, methylation, phosphorylation, and ubiquitination of histone proteins are such regulations affecting gene expression [111, 112]. Histone modifications and histone regulatory enzymes are well known to have a pivotal role in cutaneous wound healing and scarring [113]. For instance, histone H3K27me3 demethylase JMJD3 is required for the proper function of keratinocytes in wound healing. JMJD3 in cooperation with NF-kB induces the expression of inflammatory, MMPs, and mitogenic growth factor genes at the wound edge. Using in vitro and in vivo mouse skin wound models, Na and colleagues showed that the increased level of JMJD3 in the wounded environment promotes keratinocyte migration, while JMJD3 depleted cells are associated with delayed wound closure and aberrant repair [114]. Moreover, histone deacetylases (HDACs) such as HDAC-2 and sirtuin-1 (SIRT1) are the other histone-modifying enzymes reported to be involved in cutaneous scarring, thus are promising targets for scar management [115, 116, 117]. Diao et al. [116] depicted that trichostatin A, an inhibitor of HDACs, attenuates hypertrophic scarring in the rabbit ear model through decreased expression of ECM proteins including fibronectin and type I collagen, and also causes the scarred tissue to look more similar to normal appearing scar [116]. This inhibitor induces cell apoptosis and reduces cell growth and collagen synthesis in keloid fibroblasts as well [115]. It seems that trichostatin A plays a role via the repression of Sp1 activity and overexpression of secreted frizzled-related protein 1 (SFRP1) [115, 118]. On the contrary, Bai et al. [119] represented that overexpression of SIRT1 via resveratrol treatment leads to more organized and thinner collagen fibers, similar to normal scars, in the mouse model of wound healing. SIRT1 reduces the expression of αSMA and type I and III collagens in hypertrophic scar fibroblasts. However, decreased expression of SIRT1 observed during hypertrophic scar formation prevents the associated anti-fibrotic effects and drives skin fibrosis [119].

In short, optimized wound healing requires the regulated patterns of DNA methylations and histone modifications, and epigenetic alterations may result in abnormal repair and scar formation. Hence, targeting epigenetic modifying enzymes provides the therapeutic opportunity to reverse these deleterious alterations. To date, several clinical trials have been performed based on the regulation of hypermethylation patterns of different genes, and several inhibitors of HDAC are already in clinical use [117, 120]. These observations indicate the relatively smooth pathway of such strategies from bench to bedside. However, an important challenge to be carefully considered is whether up- or downregulation of epigenetic modifying enzymes could affect the expression of oncogenes or tumor suppressor genes in adjacent sequences. Further studies are thus required particularly in skin-related fibrosis to provide an effective and safe scar management approach(es).

### Noncoding RNAs in Scar Formation

Noncoding RNAs make up the vast majority of transcripts in the genome lacking the capacity to translate into proteins. They are known as crucial regulators of cellular physiology and pathology and represent great promises for the development of diagnostic and therapeutic strategies [121, 122]. Noncoding RNAs have the potential to normalize aberrant genetic networks, as they usually have several gene targets. Additionally, therapies based on noncoding RNAs allow the target genes to be modulated without genomic manipulation, as opposed to some other gene therapies. In contrast to many traditional treatments, noncoding RNAs are natural molecules in the cells and function in a targeted manner; therefore, they can be safer and/or more efficacious in treating scars [123]. MicroRNAs and lncRNAs are noncoding ribonucleic acids emerging as new therapeutic tools through epigenetic regulations as discussed below.

#### MicroRNAs as the Regulatory Switches during Scar Formation

**Fig. 2:**
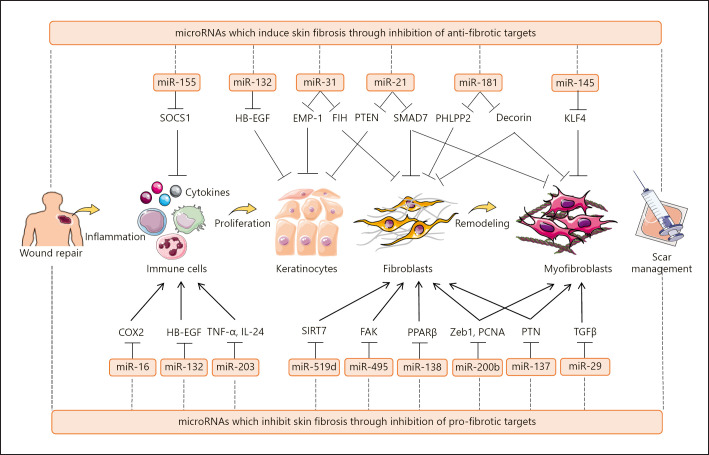
MicroRNAs as regulators of cutaneous wound healing and scar formation. MicroRNAs are key epigenetic regulators of wound healing since they can target several genes simultaneously. Dysregulation of the microRNA network in either overexpression of pro-fibrotic microRNAs, such as microRNA-155, microRNA-132, microRNA-31, microRNA-21, microRNA-181, and microRNA-145, or downregulation of anti-fibrotic microRNAs, including microRNA-16, microRNA-203, microRNA-519d, microRNA-495, microRNA-138, microRNA-200b, microRNA-137, and microRNA-29, propels the wounds to heal aberrantly, resulting in excessive cutaneous scarring. Hence, modulating wound repair through microRNAs regulation may be an interesting approach in scar management. Arrows indicate “induction,” and blunt-ended lines indicate “inhibition.”

MicroRNAs are small noncoding RNA molecules serving as the posttranscriptional gene expression regulators, thereby modulating virtually all biological processes and developmental pathways [124, 125, 126, 127, 128]. The evidence is constantly mounting that microRNAs are capable of regulating different aspects of wound healing, and their dysregulation is linked to aberrant wound repair and scar formation. Li and colleagues [129] reported that micro­RNA-132 and microRNA-31 are principal regulators promoting keratinocyte migration and proliferation during the wound healing process. Using in vitro, human ex vivo, and mouse wound models, microRNA-132 was revealed to target heparin-binding EGF*-*like growth factor and regulate the cell cycle-related genes as well as the immune response associated genes to enhance the transition from inflammation to proliferation phases of wound healing [129]. On the other hand, microRNA-31 directly targets epithelial membrane protein 1 (EMP-1) in keratinocytes and improves wound healing [130]. MicroRNA-31 is overexpressed in hypertrophic and keloid tissues and accelerates fibroblast proliferation and invasion through the FIH/HIF-1α/VEGF signaling pathway [131, 132]. Additionally, microRNA-21 plays a critical role to enhance the expression of fibro-proliferative-related genes such as *Col1A1*, *Col3A1*, *α-SMA*, and fibronectin [133, 134]. MicroRNA-21 is upregulated in hypertrophic scar tissue, keloid epidermis, and keloid-derived fibroblasts [133, 135, 136, 137]. Li, Guo, and their colleagues showed that the inhibition of microRNA-21 reduces fibrosis in the hypertrophic scar nude mice and rabbit ear models [133, 138]. Conversely, microRNA-29b and microRNA-495 serve as anti-fibrotic noncoding RNAs. Guo et al. [139] represented that microRNA-29b is downregulated in thermal injury tissue of mice and microRNA-29b treatment suppresses collagen deposition and fibrotic gene expression in scar tissues via inhibition of the TGF-β1/Smad/CTGF signaling pathway. Similarly, Guo and colleagues [140] revealed the reduced expression of microRNA-495 in the hypertrophic scar tissue and fibroblasts. Overexpression of microRNA-495 was found to inhibit focal adhesion kinase and COLA1 expression in a rat wound model, while it promotes cell death during skin regeneration [140]. Recently, several other microRNAs have been identified including microRNA-155, −181, −145, −16, −203, −519d, −138, −200b, and −137, which affect cutaneous scarring via inhibition of pro-fibrotic or anti-fibrotic targets [134, 141, 142, 143, 144, 145, 146, 147, 148] (Fig. 2). This suggests that microRNAs have the potential to be modulated for wound repair optimization and scar management.

#### LncRNAs as Regulatory Switches during Scar Formation

**Fig. 3:**
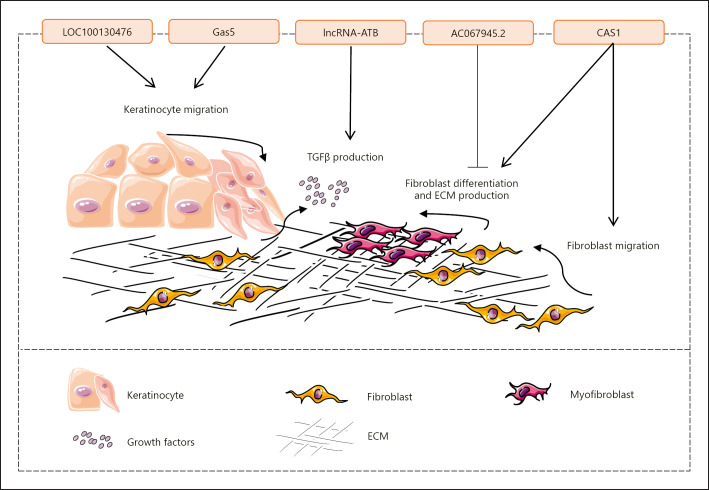
LncRNAs are regulatory RNA molecules involved in wound repair. LncRNAs are believed to regulate different aspects of wound healing. For instance, LOC100130476, Gas5, lncRNA-ATB, AC067945.2, and CAS1 affect wound healing through regulation of keratinocyte migration, growth factor production, fibroblast migration and differentiation, and ECM production. Hence, lncRNAs possess the potential to be manipulated for scar management. Arrows indicate “induction,” and blunt-ended lines indicate “inhibition.”

LncRNAs are regulatory RNA molecules containing more than 200 nucleotides involved in the modification of chromatin, production of endogenous siRNA, regulation of RNA processing, modulation of microRNA activity, and regulation of protein functions and localization [149, 150]. LncRNAs are believed to be related to the pathophysiology of wound repair (Fig. 3). Herter et al. [151] demonstrate that the expression of lncRNA LOC100130476 is reduced in keratinocytes of the human chronic wound edge. LncRNA LOC100130476 restricts the production of inflammatory chemokines by keratinocytes and enhances cell migration. Hence, its downregulation leads to impaired reepithelialization, as observed in human ex vivo wounds [151]. Moreover, lncRNAs could be targeted by drugs to improve wound healing. Using an ex vivo model for treated tissue samples obtained from the nonhealing edge of human wounds, Sawaya and colleagues [152] showed that topical mevastatin accelerates wound closure, in part through induction of lncRNA Gas5. LncRNAs are also important in cutaneous scar formation. Chen et al. [153] demonstrated that AC067945.2 is downregulated in hypertrophic scar tissues, while its overexpression promotes early apoptosis and diminishes the expression of COL1A1, COL1A2, COL3A1, and α-SMA in skin fibroblasts through VEGF regulation. On the other hand, lncRNAs such as CACNA1G-AS1 (CAS1) and lncRNA activated by TGF-β (lncRNA-ATB) affect keloid formation. CAS1 is contributed to the WNT signaling pathway and is significantly overexpressed in keloid scar compared to normal skin. CAS1 enhances the expression of the calcium channel protein, synthesis of type I collagen, and also the migration of keloid fibroblasts [154, 155]. In addition, lncRNA-ATB is overexpressed by TGF-β signaling and negatively regulates the activity of microRNA-200c resulting in the removal of associated inhibitory effect on ZNF217 transcript. Consequently, the increased expression of ZNF217 protein activates TGF-β2 transcription, which in turn exacerbates TGF-β signaling and maintains the lncRNA-ATB/microRNA-200c/ZNF217/TGF-β2 loop mediating cutaneous fibrosis [156].

Altogether, increasing published research data indicate the regulatory role of noncoding RNAs during wound repair and suggest that these players open new doors for the development of diagnostic and therapeutic strategies of wound complications. Although in comparison with lncRNAs, more studies have been accomplished on microRNAs and their functions, there are yet several challenges to bring these tiny RNAs to wound therapy. First, the expression profile of microRNAs is context-dependent and different in ex vivo and experimental models compared to that in human wounds due to different cell behaviors. Hence, confirmation of an identified microRNA as a therapeutic target is accompanied by complications. Second, the development of microRNAs as therapeutic tools necessitates efficient systems for the safe and accurate delivery of agents to specific target cells and tissues. Generally, methods based on viral and nonviral delivery are considered in research and clinical applications, of which viral deliveries are associated with concerns such as toxicity and immunogenicity. Therefore, nonviral delivery systems including modified oligonucleotides, exosomes, lipid, and polymer-based methods, and engineered nanoparticles have recently gained great attention [157], albeit requiring further optimizations in the therapeutic landscape. Third, microRNAs have the potential to target several genes and signaling pathways in a complex wound healing process. Regarding this, modulation of microRNAs should be investigated in terms of inadvertent dysregulation of genes. Last but not least, in wound modeling for RNA-based modification, most studies have been focused on animal skin that is structurally different from human skin, indicating that translation of findings may be challenging [101]. Therefore, the models further mimicking human skin such as a three-dimensional in vivo tissue-like environment provided from organoid culture and microfluidic system humanized animal models and engineered skin models are needed to extend the results into the human wound dysregulations.

## Conclusion

**Fig. 4:**
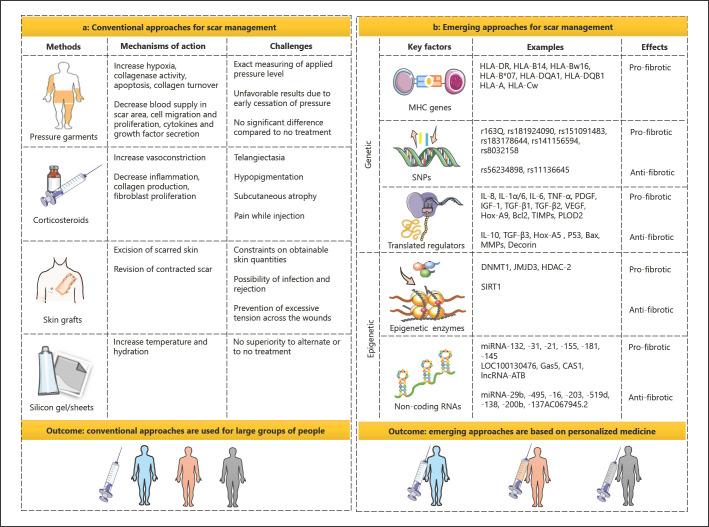
Conventional and emerging therapeutic approaches for scar management. **a** The conventional approaches for scar management include pressure garments, corticosteroids, skin grafts, and silicone gels and sheets affecting wound healing and scar formation through different mechanisms of action. However, they are not targeted and efficient enough due to associated challenges. Conventional strategies are also used for large groups of people. Therefore, (**b**) new methods based on the optimization of wound healing procedure in terms of genetic and epigenetic regulations are emerging to diminish or prevent dermal fibrosis. MHCs and SNPs, genes coding for key regulators such as cytokines and growth factors, DNA methylation signatures, histone modification patterns and related enzymes, and noncoding RNAs interact and cooperate as a complex network affecting cell proliferation, differentiation, and ECM synthesis. Deregulation of the genetic and epigenetic network leads to aberrant behavior of different cells driving excessive fibroblast proliferation and differentiation, and consequently cutaneous fibrosis. Thus, these regulators can be identified via genetic and epigenetic screening and targeted using precision medicine strategies for scar management.

Wound healing is a complex process, in which an orchestrated array of biological and molecular events occurs to promote skin regeneration. However, any abnormalities in wound healing lead to reparative responses driving the normal repair process to the formation of thick, painful, and itchy scars accompanied by esthetic and functional complications. Nowadays, the majority of the strategies used for scar treatment are not efficient and specific enough. The effective application of pressure garments highly depends on the applied pressure levels, and measuring the exact pressure level is difficult and controversial. A recent study found no significant difference between pressure garments and the no-treatment group in patients with large burn scars. Silicone gels and sheets were also found to not be superior to the no-treatment group in preventing or treating scar tissues. Furthermore, their various side effects such as subcutaneous atrophy and pain, and constraints on obtainable skin quantities for skin grafts have restricted their clinical utility. Hence, developing more effective and targeted therapies for preventing the deposition of excessive fibrous tissue is of critical importance. This purpose is achieved through proper knowledge about the regulatory mechanisms of wound healing and scarring. Given the importance of genetic and epigenetic differences in physiologic or pathologic wound repair, we highlighted the diversity of the MHC genes and SNPs determining the susceptibility to scar formation to present the scar management techniques through the establishment of prognosis and prevention strategies and subsequently personalized medicine. For this achievement, genetic screening to discover the variations and understanding their roles in wound repair is necessary, which requires available tools and technologies, and well-powered studies in the future. Moreover, key insights were obtained from the analyses of gene dysregulations involved in inflammation, granulation tissue formation, apoptosis, and ECM remodeling suggesting genetic interventions to improve wound healing and block skin fibrosis. However, the selection of targeted cells, spatiotemporal regulation of targeted mediators due to their dual activities, the proper level of manipulations, and the application of an appropriate strategy for safe, targeted, and efficient gene delivery were discussed as challenges that need to be addressed. Finally, we presented epigenetic alterations including DNA methylations, histone modifications, and noncoding ribonucleic acids such as microRNAs and lncRNAs serving as crucial regulatory switches in the wound healing process. The regulatory epigenetic molecules were indicated to be promising therapeutic tools for scar management, although their associated exact roles need to be further explored in terms of inadvertent dysregulation of genes. The models that resemble human cutaneous wounds were also mentioned to be required in preclinical settings to pave the way for more effective scar management (Fig. 4). Altogether, clinical trials based on the application of IL-10, TGF-β, siRNA STP705, and RXI-109, regulation of hypermethylation patterns, and histone modifications are underway, representing the therapeutic potential of genetic and epigenetic approaches for optimization of wound healing and scar management. However, successful application of these emerging strategies requires the advancements in available tools and technologies for discovery, delivery, and controlled manipulation of genetic and epigenetic regulators. This helps overcome the associated challenges and opens new doors for management of cutaneous fibrosis.

## Conflict of Interest Statement

The authors have no conflict of interest.

## Funding Sources

This study was funded by a grant provided by Royan Institute.

## Author Contributions

The conception and design of the review were provided by Sara Amjadian and Parvaneh Mohammadi. The manuscript was drafted by Sara Amjadian. The writing of the manuscript was revised and directed by Parvaneh Mohammadi and Sharif Moradi.

## Figures and Tables

**Table 1:** The important genes during different phases of wound healing and scar formation

Gene/factor	Role in		Effects	References
IL-8	Induction Inhibition	Inflammation; recruitment of immune cells and fibroblasts; hemostasis of epidermis; proliferation of keratinocytes; production of MMP-9 in keratinocytes; angiogenesis −	Pro-fibrotic	[33–35]

IL-1α	Induction Inhibition	Inflammation; activity of keratinocytes, fibroblasts, and endothelial cells; deposition of collagen Restoration of the skin architecture	Pro-fibrotic	[37,38]

IL-1β	Induction Inhibition	Expression of decorin; activity and homing of PBMC; function of CXCR4-CXCL 12 axis; proliferation of fibroblasts Restoration of the skin architecture	Pro-fibrotic	[37, 39]

IL-6	Induction Inhibition	The pro-inflammatory function of immune cells; timely resolution of wound healing −	Pro-fibrotic	[41]

TNF-α	Induction Inhibition	Inflammation; EMT; expression of MMPs; apoptosis TGF-β and CTGF signaling; expression of αSMA −	Pro-fibrotic or anti-fibrotic	[42–45, 95]

IL-10	Induction Inhibition	Physiological wound healing; organization and maturation of ECM; production of hyaluronic; angiogenesis Activation and migration of immune cell; expression of TGF-β; deposition of excessive collagen; expression of αSMA	Anti-fibrotic	[46–49]

PDGF	Induction Inhibition	Chemotaxis of dermal fibroblasts; proliferation of keratinocyte and fibroblast; synthesis of ECM components; degradation of the old collagen fibers; angiogenesis −	Pro-fibrotic	[51–54]

IGF-1	Induction Inhibition	The proliferation of fibroblast; synthesis and organization of ECM; renewal activity of keratinocytes; angiogenesis; wound contracture Apoptosis of keratinocytes	Pro-fibrotic	[55,59]

TGF-β1;TGF-β2	Induction Inhibition	Migration of inflammatory cells, keratinocyte, and fibroblasts; proliferation of cells; expression and organization of ECM; expression of TIMPs and αSMA; angiogenesis; trans-differentiation of fibroblasts Expression of MMPs; apoptosis	Pro-fibrotic	[34, 61, 62, 68]

TGF-β3	Induction Inhibition	Expression of MMPs; degradation of collagen fibers Differentiation of fibroblasts; deposition of collagen type 1	Anti-fibrotic	[34, 62, 68]

P311	Induction Inhibition	Translation of TGF-β isoforms; proliferation and migration of cells; expression of COL1A1 and αSMA; tensile strength of newly formed tissue and normal scar formation −	Pro-fibrotic	[69, 70]

VEGF	Induction Inhibition	Recruitment of mast cells and other inflammatory cells; proliferation and migration of endothelial cells and keratinocytes; angiogenesis −	Pro-fibrotic	[71, 74]

Hox-A9	Induction Inhibition	Transcription of VEGF −	Pro-fibrotic	[72]

Hox-A5	Induction Inhibition	Expression of antiangiogenic genes Expression of pro-angiogenic genes	Anti-fibrotic	[72]

p53; Bax	Induction Inhibition	Programmed cell death; organization of collagen −	Anti-fibrotic	[77]

Snail-2; SFRP2	Induction Inhibition	Synthesis of collagen Programmed cell death; organization of collagen	Pro-fibrotic	[76]

MMP-1 (collagenases I)	Induction Inhibition	Migration and reepithelialization of keratinocyte; degradation of fibrillar collagens −	Anti-fibrotic	[78, 80]

MMP-2 (gelatinases)	Induction Inhibition	Degeneration of gelatin segments and other ECM proteins; prolonged remodeling events −	Pro-fibrotic or anti-fibrotic	[79, 80, 86]

MMP-3	Induction Inhibition	Degradation of collagen type III; wound contraction by fibroblasts −	Anti-fibrotic	[78, 80]

MMP-8 (collagenases II)	Induction Inhibition	Degradation of fibrillar collagens −	Anti-fibrotic	[78, 80]

MMP-9 (gelatinases)	Induction Inhibition	Migration and reepithelialization of keratinocytes; degeneration of gelatin segments and other ECM proteins; early events of wound repair	Anti-fibrotic	[78–80]

MMP-13 (collagenases III)	Induction Inhibition	Degradation of fibrillar collagens	Anti-fibrotic	[78, 80]

TIMP-1	Induction Inhibition	Proliferation of keratinocytes Degradation of ECM; activity of MMP-1 and −2; apoptosis	Pro-fibrotic	[80, 81, 83]

TIMP-2	Induction Inhibition	Migration of keratinocytes Degradation of ECM; activity of MMP-1 and −2; apoptosis	Pro-fibrotic	[81, 82]

TIMP-3	Induction Inhibition	− Degradation of ECM; activity of MMP-1, −2, −3, −9, −13; inflammation; angiogenesis	Pro-fibrotic	[80, 81]

PLOD2	Induction Inhibition	Pyridinoline cross-linkage of collagen; accumulation of collagen Degradation of collagen	Pro-fibrotic	[87, 88]

Decorin	Induction Inhibition	Reorganization of collagen fibers; tensile strength of the skin TGF-β signaling	Anti-fibrotic	[6, 61, 89]

SFRP2, secreted frizzled-related protein 2.
